# Prize Essay

**Published:** 1882-02-20

**Authors:** 


**Prize Essay.**—The Committee on Prize Essays for the Kentucky State Medical Society has decided to offer fifty dollars ($50) for the best essay embodying the results of original experimental research, or original clinical observation on the nature, mode of propagation, pathology, and treatment of scarlatina.

1. Competing essays must be the composition of, and in the handwriting of the authors, who must be members of the Kentucky State Medical Society.

2. They must be marked by a motto or character, accompanied by a sealed envelope, bearing the same motto or character, enclosing the author's name

3. They must be sent to the chairman of the committee before the 15th day of March, 1882.

The committee may reject any or all essays presented. In case of award, the successful essay shall be read to the Society on the morning of the second day of the annual meeting, after which the chairman of the committee shall open the sealed envelope, make known the name of the author, and publicly award the prize.

DUDLEY S. REYNOLDS, M. D., Chairman.
HENRY M. SKILLMAN, M. D.,
A. R. MCKEE, M. D.,
DAVID W. YANDELL, M. D.,
CHARLES H. TODD, M. D.

